# A Case Report on Diagnostic Approach of a Complex Clinical Scenario: Differentiation Between Coronary Artery Disease and Apical Hypertrophic Cardiomyopathy as a Cause of Recurrent Debilitating Angina

**DOI:** 10.7759/cureus.14496

**Published:** 2021-04-15

**Authors:** Shanan Mahal, Tushar Tarun, Sorabh Datta, Kul Aggarwal

**Affiliations:** 1 Department of Internal Medicine, Baptist Health–University of Arkansas for Medical Sciences, North Little Rock, USA; 2 Division of Cardiovascular Medicine, University of Missouri, Columbia, USA

**Keywords:** echocardiogram, apical hypertrophic cardiomyopathy, epicardial coronary artery disease, cardiac magnetic resonance imaging, midventricular obstruction with cavity obliteration, coronary artery disease, left ventricular hypertrophy, beta blockers, apical myectomy, calcium channel blockers

## Abstract

Apical hypertrophic cardiomyopathy (ApHCM) is a clinical challenge when presenting with symptoms of angina plus shortness of breath. An appropriate diagnosis of concurrent coronary artery disease (CAD) is needed for proper diagnosis, risk stratification, and management. We present a case of a 64-year-old gentleman with a history of ApHCM and CAD with previous percutaneous intervention presenting with recurrent angina. A repeat coronary angiography showed no new obstructive lesions. A stress cardiac magnetic resonance imaging was performed, which showed perfusion defect in the apex with apical scarring likely secondary to microvascular disease. The patient was managed medically with the improvement of symptoms. Diagnosis and management of CAD in patients with ApHCM are challenging. Multiple diagnostic modalities may be required for delineating the underlying pathology. Patients should be managed initially with medications. If symptoms are not controlled with medical management, a heart team approach with referral to an advanced center experienced in apical myectomy should be considered.

## Introduction

One of the rare variants of hypertrophic cardiomyopathy is apical hypertrophic cardiomyopathy (ApHCM) which mainly affects the left ventricular apex. Most patients with this morphology present with midventricular obstruction rather than left ventricular outflow tract obstruction (LVOTO). Based on some research studies, it has been found that ApHCM is mostly prevalent in the East Asian population [1,2]. ApHCM is a clinical challenge when presenting with symptoms of angina plus shortness of breath. An appropriate diagnosis of concurrent CAD is needed for proper diagnosis, risk stratification, and management.

## Case presentation

**Figure 1:**
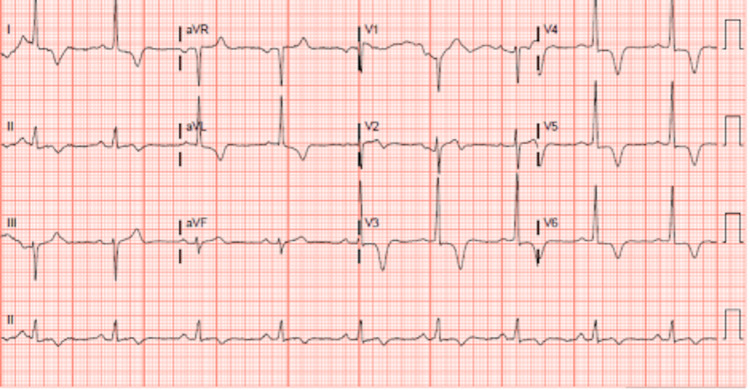
ECG at the time of clinic visit showing LV hypertrophy with inverted T waves in V3, typical of ApHCM. ECG - electrocardiogram; LV - left ventricle; ApHCM - apical hypertrophic cardiomyopathy

A 64-year-old male with a past medical history of coronary artery disease (CAD) and ApHCM presented to the cardiology clinic with recurrent exertional chest pain. He had undergone coronary angiography 10 months previously for angina, which documented 80% stenosis of the first posterolateral branch of the right coronary artery (RCA) with the subsequent percutaneous intervention of the lesion. Symptoms of chest pain initially improved but recurred in the four months before his clinic follow-up and were described as central non-radiating chest discomfort on minimal exertion that typically resolved with rest. Repeat electrocardiogram (ECG) showed left ventricular (LV) hypertrophy with deep T wave inversion in V3, unchanged from previous ECG and attributed to known ApHCM (Figure 1).

**Figure 2:**
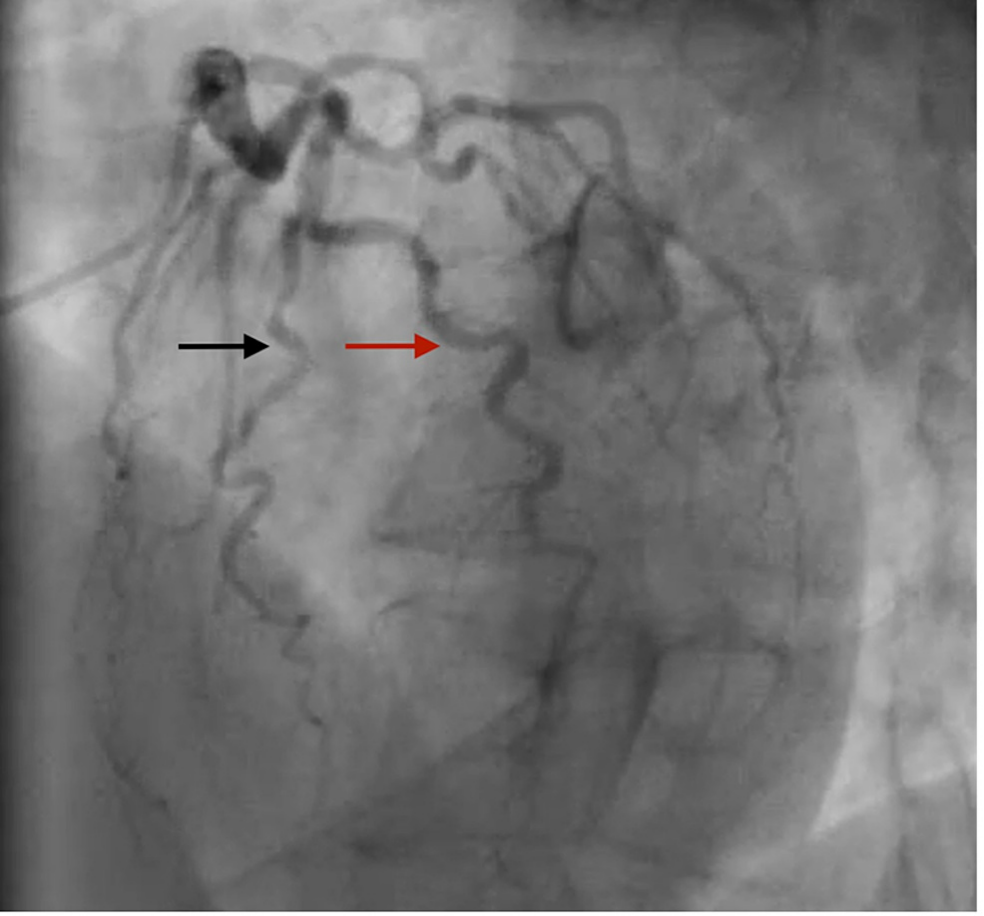
Cardiac catheterization, RAO cranial view, showing 30% stenosis of the LAD coronary artery (black arrow) and the diagonal branch of the LAD coronary artery (red arrow); the LAD is small compared to the diagonal branch. LAD - left anterior descending; RAO - right anterior oblique

**Figure 3:**
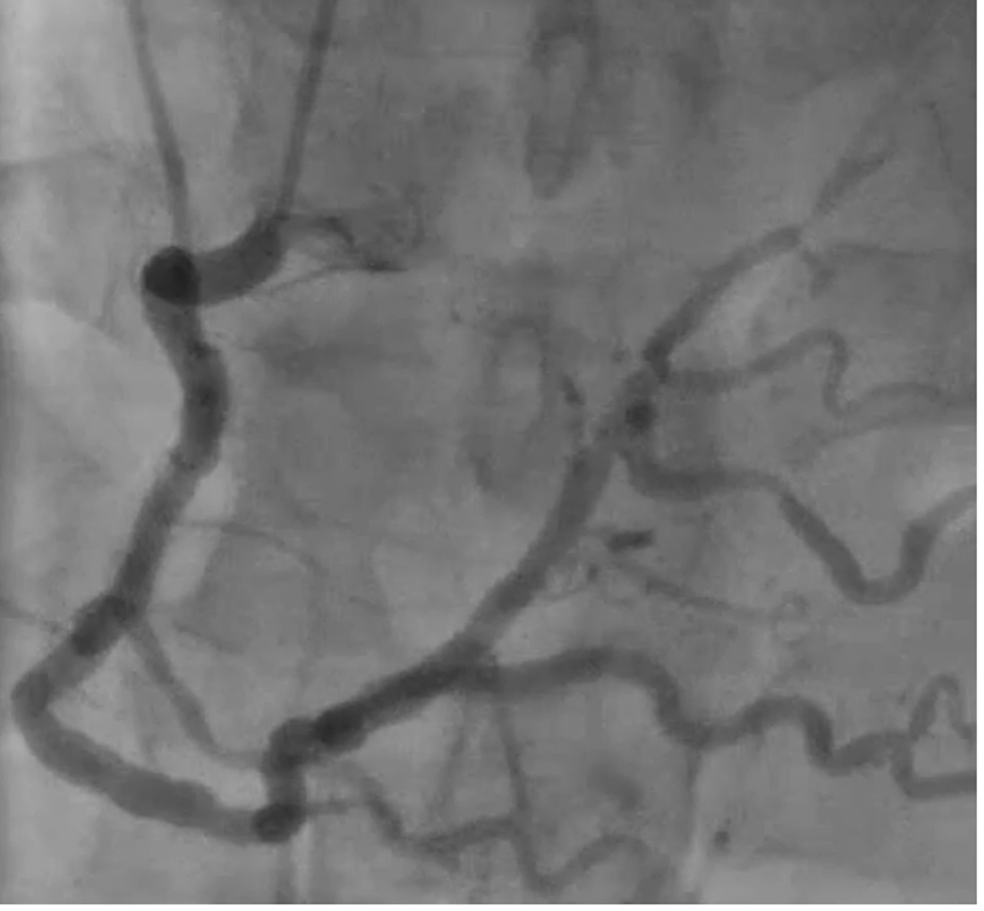
Cardiac catheterization, AP cranial view, showing no significant disease of RCA. RCA - right coronary artery; AP - anterior-posterior

Due to concerns for restenosis and/or new obstructive disease, repeat coronary angiography was performed and showed no evidence of restenosis of the stent nor new disease. The left anterior descending coronary artery (LAD) was noted to have a mild diffuse plaque with maximal 30% stenosis and to be shorter compared to the diagonal (Figure 2). RCA showed a patent stent with good flow (Figure 3).

**Figure 4:**
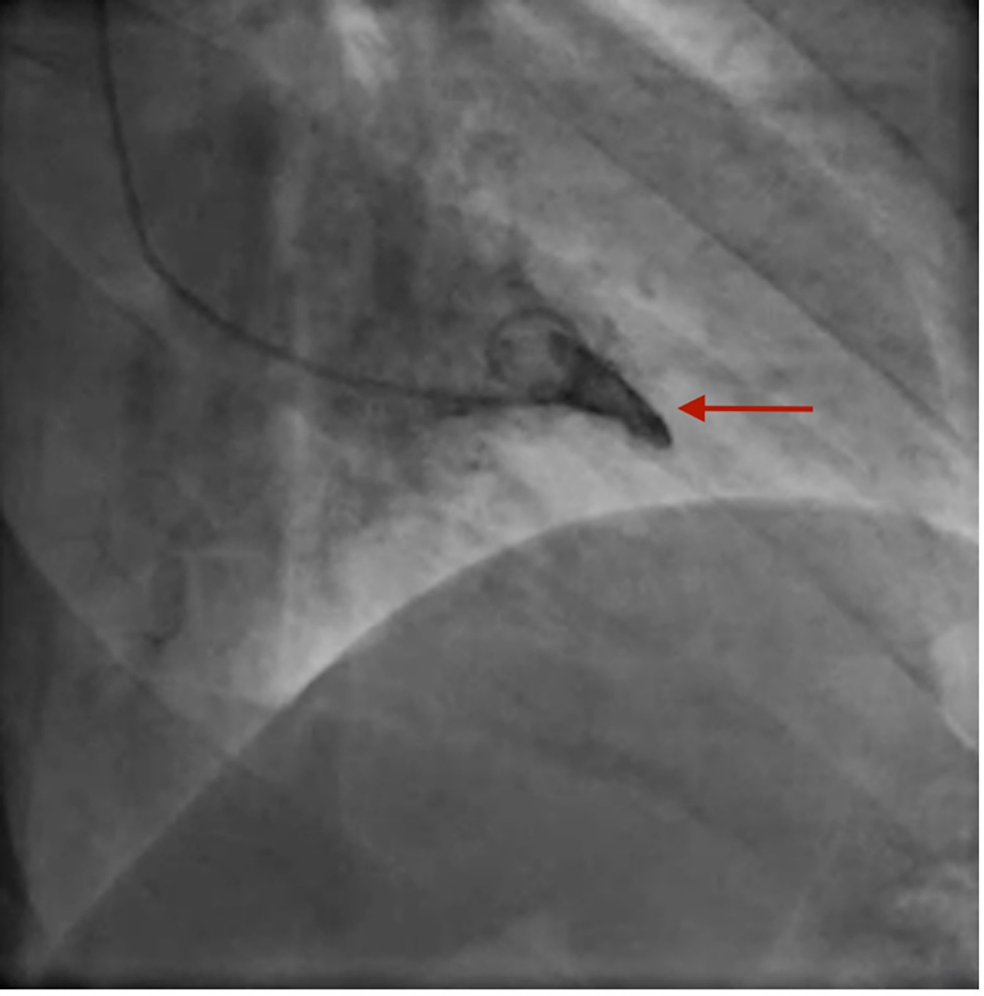
Left ventriculogram showing the ace of spades pattern (red arrow).

Left ventricular end-diastolic pressure was elevated (21 mmHg), and the left ventriculogram showed typical findings of ApHCM (Figure 4).

**Figure 5:**
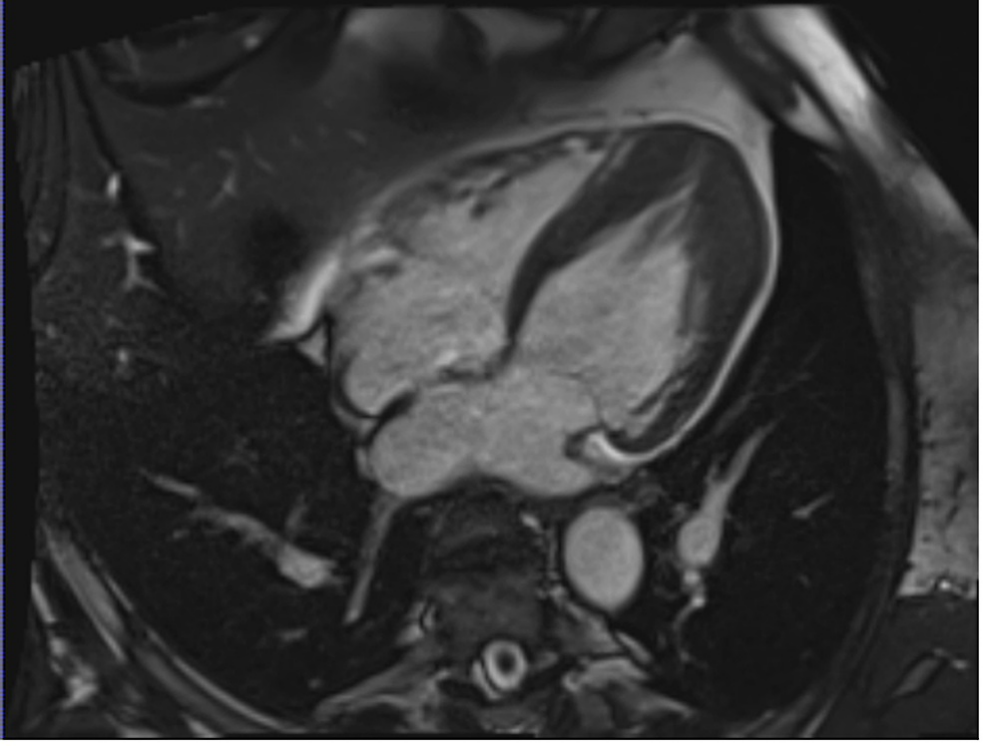
Cine-cardiac MRI showing severe apical hypertrophy, with a maximum thickness of 1.8 cm. MRI - magnetic resonance imaging

**Figure 6:**
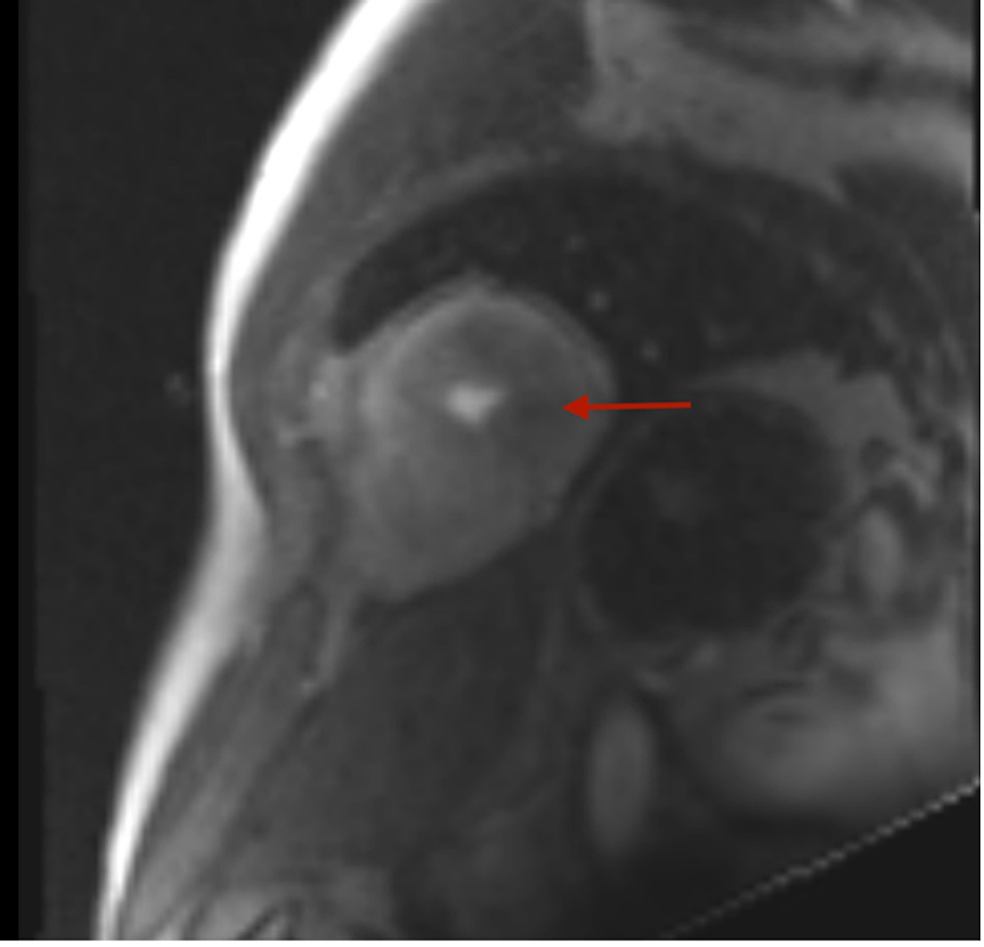
Cardiac stress perfusion MRI showing perfusion defects in the LV. MRI - magnetic resonance imaging; LV - left ventricle

**Figure 7:**
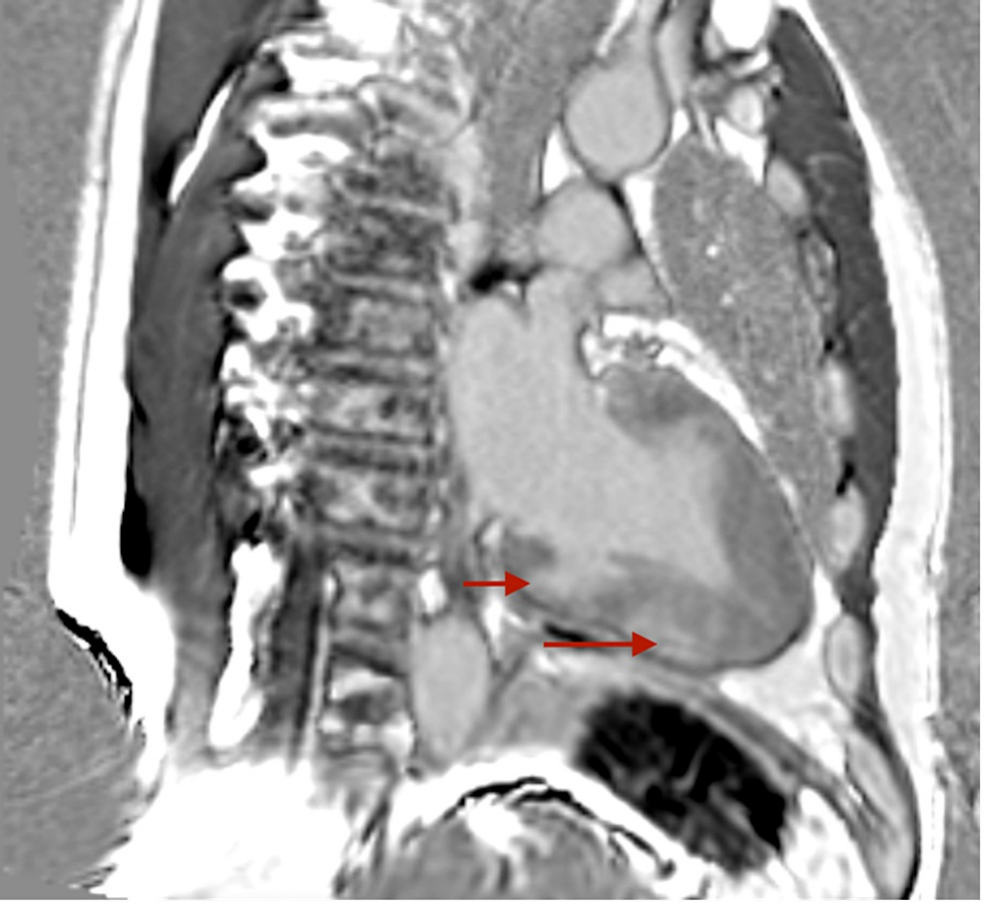
Cardiac MRI showing delayed gadolinium enhancement with red arrows showing patchy scarring of the LV apex. MRI - magnetic resonance imaging; LV - left ventricle

Subsequent stress cardiac magnetic resonance imaging (MRI) confirmed severe apical hypertrophy (Figure 5) and multiple perfusion defects in the mid and distal LV myocardium, consistent with the diagnosis of ApHCM (Figure 6). Moreover, delayed gadolinium enhancement showed patchy scarring of the LV apex (Figure 7).

Given MRI findings, his angina symptoms were attributed to microvascular ischemia of the apex secondary to severe ApHCM. The patient was given pharmacological treatment with beta-blockers (BB) and calcium channel blockers (CCB) and showed subsequent improvement.

## Discussion

ApHCM is a variant of hypertrophic cardiomyopathy (HCM) characterized by isolated hypertrophy of the LV apex. ApHCM accounts for 3-10% of all HCM in the United States compared to 25% of all HCM cases in Japan [1]. Although most cases are asymptomatic and have a good prognosis, patients with concurrent CAD have a worse prognosis [3-5]. Regarding the pathophysiology of ApHCM, it can present with or without features of midventricular obstruction with cavity obliteration (MVOCO), as well as with and without apical aneurysm formation [6]. The resultant diastolic dysfunction leads to dyspnea and can cause left atrial dilation, increasing the risk of atrial fibrillation [6,7]. LV hypertrophy can also cause myocardial ischemia due to demand-perfusion mismatch. Some features of intramural coronary artery narrowing with the resulting small vessel disease without the underlying atherosclerotic disease are also present in the ApHCM [6]. In a series of 208 patients reported from China with a diagnosis of ApHCM, presenting symptoms were predominantly chest discomfort and chest pain (91.8%), palpitations (30.8%), dyspnea (10.6%), dizziness (9.6%), and presyncope or syncope (7.2%) [8].

The evaluation includes an ECG and an echocardiogram, which show high QRS voltage and symmetrical deep T waves in the left precordial leads and increased apical wall thickness of more than 15 mm [9]. Coronary angiography aids the diagnosis of concurrent epicardial coronary artery disease; left ventriculogram also shows a typical ace of spades pattern. Cardiac MRI has the unique capability to define myocardial structure and function, and it is useful for diagnosis and risk stratification. Increased apical late gadolinium enhancement predicts worse microvascular disease and increased cardiovascular mortality in several studies [10-13]. Myocardial perfusion defects can be seen on single-photon emission computed tomography (SPECT), which might be secondary to non-ischemic versus ischemic processes [6]. An increase in apical tracer uptake and spade-like deformity of the LV, referred to as a solar polar map pattern, is also demonstrated by SPECT [14].

Medical management is similar to therapy for HCM. BBs are the backbone of the treatment [6]. CCBs are the additional therapy for patients with persistent symptoms despite the BB therapy. There is some evidence of the benefit of other therapies, such as angiotensin-converting enzyme inhibitors; however, further investigation is ongoing for different therapies [5]. In patients with ongoing symptoms despite medical management, there may be a role of surgical treatment with resection of hypertrophied apical muscle [7].

**Figure 8:**
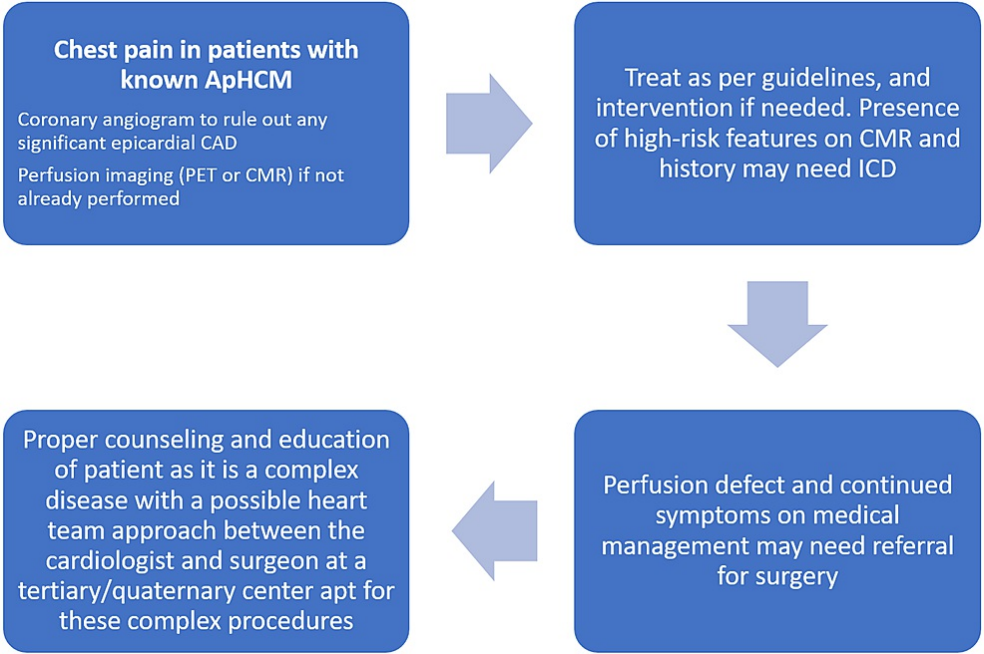
Algorithm addressing the evaluation of ApHCM. ApHCM - apical hypertrophic cardiomyopathy; CAD - coronary artery disease; PET - positron emission tomography; CMR - cardiac magnetic resonance imaging; ICD - implantable cardioverter defibrillator

Some patients have shown improved symptoms with the novel surgical technique of apical myectomy [15,16]. Alcohol septal ablation has shown limited benefits in ApHCM patients compared to the HCM group of patients, although it can be considered in patients with MVOCO as they are generally considered to be high risk for surgery [6]. A proposed strategy for the evaluation of ApHCM is illustrated in Figure 8.

## Conclusions

Patients with ApHCM can present with angina, which poses a diagnostic dilemma of whether symptoms are caused by concomitant coronary disease. Careful patient evaluation with angiography and perfusion imaging helps delineate the underlying pathology and permits more specific treatment and risk stratification because patients with concurrent CAD have a worse prognosis.
